# Publishing in Open-Access Journals: Potential Pitfalls

**Published:** 2013-07-01

**Authors:** Pamela Hallquist Viale

As most of you know, at the Journal of the Advanced Practitioner in Oncology (JADPRO) we do not charge a fee to US readers who subscribe to the journal. We do not charge potential authors to have their work reviewed by our blind review process, nor do we charge for publication. Our publisher is the respected Harborside Press, also the publisher of the Journal of the National Comprehensive Cancer Network and TheASCO Post. I believe JADPRO publishes high-quality, relevant articles that inspire and educate the advanced practitioner working in the field of oncology.

The open-access publishing phenomenon started approximately 10 years ago, with the introduction of respected journals published by the Public Library of Science (Kolata, 2013). Open access allows access to many helpful, scholarly articles (such as the professional papers available via PubMed Central). It’s a boon to readers who can’t afford separate subscriptions to a myriad of scientific journals but need the information for practice and education.

## Predatory Publishers

The term "predatory publisher" refers to a publishing firm that exploits the open-access model to one that requires the author to pay (Beall, 2012). Predatory publishers using the open-access model often charge authors a fee to publish without quality control measures such as blinded peer review (Haug, 2013). Many of these publishers are not up front about their publication. It may be difficult to find out exactly where the publisher’s headquarters are located; they are often located in third-world economies, such as Pakistan or Nigeria (Beall, 2012).

Predatory publishers may send out "spam" emails requesting potential authors to send manuscripts for publication. These emails are usually easy to pick out, but that’s not always the case. Frequently, there are misspellings or obvious grammatical errors (Beall, 2012). A recent spam email request for a paper had suspicious language such as "your active and enthusiastic submission of manuscript which will be published under respective special issue title you choose for this glorious year." It may be difficult to identify the members of the editorial board. Often the salutation is incorrect on the email; for example, I am usually labeled a PhD or an MD, rather than my correct title of nurse practitioner or master’s prepared registered nurse. Some of these invitations even "promise" to publish an article within a very short time period—sight unseen!

## The "Author Pays" Model

The "author pays" model has indeed been used successfully in many respected publications; it’s not completely verboten. Due to the volume of submissions received by ASCO’s Journal of Clinical Oncology, potential authors must pay a fee ($60.00) to be reviewed (American Society of Clinical Oncology, 2013). A friend of mine was recently charged a hefty fee for his accepted publication in the journal Ecology. As a graduate student, his excitement at being published was quickly overshadowed by the publication bill ($75.00 per printed page); fortunately, his university was willing to pick up the charge (Ecology, 2013). Authors publishing in Dovepress journals are charged a processing fee to pay for editorial and production costs, although a fund exists to cover costs for authors from less developed countries (Dovepress, 2013). These are just a few examples from the world of scientific publishing.

The "author pays" model is probably here to stay, and open-access publishing has many positive qualities. However, predatory publishers should be approached warily. These requests from publishers are frequently for the specific purpose of acquiring additional income, without adding substantially to professional education or scholarship (Haug, 2013). A recent editorial on predatory publishing in TheNew York Times discussed the experiences of scientists who were recruited to speak at a conference; once they appeared, they found out that not all the speakers were necessarily experts in the field and all had been recruited by email (Kolata, 2013). The speakers who ended up making a presentation were later charged a significant fee for the "privilege" of speaking (Kolata, 2013).

## Beall’s List of Predatory Publishers

Jeffrey Beall is an academic research librarian at the University of Colorado in Denver with a keen interest in the concept of predatory publishers. Mr. Beall has followed questionable publishers and journals and developed a list of open-access predatory publishers and journals. You can access this list at http://scholarlyoa.com/individual-journals/. Beall has also listed his criteria for determining whether a journal is potentially predatory or not.

## The Need for Professional Publishing

As the editor-in-chief of JADPRO, I am excited to offer our readers substantial, expert articles approved by our blind review process and professional editorial board members. JADPRO does not charge a fee for either the review or publication of an accepted paper. All content is rigorously evaluated by our review board in terms of relevance, appropriateness, and academic scholarship. I believe our journal offers our readers vetted quality information via the "open-access" model. Professional publishing is an excellent way to share one’s knowledge with others. We welcome potential authors to submit appropriate manuscripts to JADPRO. No money required—bring only your commitment to excellence and your passion for what you do.
